# Key site residues of pheromone-binding protein 1 involved in interacting with sex pheromone components of *Helicoverpa armigera*

**DOI:** 10.1038/s41598-017-17050-5

**Published:** 2017-12-04

**Authors:** Kun Dong, Hong-Xia Duan, Jing-Tao Liu, Liang Sun, Shao-Hua Gu, Ruo-Nan Yang, Khalid Hussain Dhiloo, Xi-Wu Gao, Yong-Jun Zhang, Yu-Yuan Guo

**Affiliations:** 1 State Key Laboratory for Biology of Plant Diseases and Insect Pests, Institute of Plant Protection, Chinese Academy of Agricultural Sciences, Beijing, 100193 China; 2 Department of Entomology, China Agricultural University, Beijing, 100193 China; 3 College of Science, China Agricultural University, Beijing, 100193 China; 4 Key Laboratory of Tea Biology and Resources Utilization, Ministry of Agriculture, Tea Research Institute, Chinese Academy of Agricultural Sciences, Hangzhou, 310008 China; 5 Department of Entomology, Faculty of Crop Protection, Sindh Agriculture University Tandojam, Tandojam, Pakistan

## Abstract

Pheromone binding proteins (PBPs) are widely distributed in insect antennae, and play important roles in the perception of sex pheromones. However, the detail mechanism of interaction between PBPs and odorants remains in a black box. Here, a predicted 3D structure of PBP1 of the serious agricultural pest, *Helicoverpa armigera* (HarmPBP1) was constructed, and the key residues that contribute to binding with the major sex pheromone components of this pest, (*Z*)-11- hexadecenal (*Z*11-16:Ald) and (*Z*)-9- hexadecenal (*Z*9-16:Ald), were predicted by molecular docking. The results of molecular simulation suggest that hydrophobic interactions are the main linkage between HarmPBP1 and the two aldehydes, and four residues in the binding pocket (Phe12, Phe36, Trp37, and Phe119) may participate in binding with these two ligands. Then site-directed mutagenesis and fluorescence binding assays were performed, and significant decrease of the binding ability to both *Z*11-16:Ald and *Z*9-16:Ald was observed in three mutants of HarmPBP1 (F12A, W37A, and F119A). These results revealed that Phe12, Trp37, and Phe119 are the key residues of HarmPBP1 in binding with the *Z*11-16:Ald and *Z*9-16:Ald. This study provides new insights into the interactions between pheromone and PBP, and may serve as a foundation for better understanding of the pheromone recognition in moths.

## Introduction

Pheromones perception is crucial for insects to seek out sexual partners^1^. For Lepidoptera species, sex pheromones blends are normally produced at accurate proportion and emitted by females to attract conspecific males for mating^2,3^. Such specific perception benefits from male moths’ sophisticated olfactory system including numerous antennal sensilla, especially the sensilla trichodea, which are sensitive to different sex pheromone components^4,5^. The pheromone detection in male moths is initiated when pheromone molecules enter the lymph of trichoid sensilla through multipores^2,6^ and it is widely accepted that several different groups of olfactory proteins, such as pheromone-binding proteins (PBPs), chemosensory proteins (CSPs), sensory neuron membrane proteins (SNMPs), odorant receptors (ORs) and ionotropic receptors (IRs) are involved in the process of pheromone detection^7,8^.

Pheromone binding proteins are small (15–17 kDa) water-soluble proteins which present in the sensillar lymph with extremely high concentrations (up to 10 mM)^9,10^. These proteins are thought to solubilize, capture and transport hydrophobic pheromone molecules across the aqueous sensillar lymph to the pheromone receptors (PRs)^11,12^. The first *PBP* gene was identified in the silkworm, *Antheraea polyphemus* ^13^, then their orthologous genes have been identified in many Lepidoptera species^14^. Further research revealed that PBPs may specifically recognize distinct pheromone components and enhance the sensitivity of PRs in response to pheromones^15,16^. Because of the high sensitivity to pheromone components, PBPs are often served as the molecular targets to design the attractants of moths or other insect species^17,18^.

It is well accepted that insect PBPs play important roles in pheromone perception^7,9^. However, the detail interaction mechanism between pheromones and PBPs is still unknown. Many three-dimensional (3-D) structures of insect PBPs have been solved in the crystal forms or in solution since the structure of BmorPBP/bombykol complex was reported^19–23^. Most insect PBPs exhibit series of identical structure characteristics including six or seven α-helices, three strictly conserved disulfide bridges, and a hydrophobic binding pocket. However, structure diversity is also observed and such differences make insect PBPs show different cavity shapes and openings to accommodate distinct ligands^19,23–27^. Various studies suggested that lepidopteran PBPs existed pH-dependent conformational change associated with significant decrease in affinity at low pH values^18,19,21,22,28–31^. The C-terminals of moth PBPs fold into an additional α-helix and enter the binding pocket to occupy the corresponding pheromone-binding sites at acid pH, whereas at neutral pH, the additional helix withdraws from the binding pocket and made it available for pheromone binding^19,24^. Other insect PBPs with short C-terminals, such as the LmaPBP in cockroach, could not form the additional helix but make a lid to cover the binding pocket, and such ‘lid’ would also affect the binding between PBPs to ligands^23^. All the research revealed that insect PBPs own diverse mechanisms in ligand binding and release, and such mechanisms relate closely to the structures of PBPs. It also suggested that the structural study at molecular level should be helpful in understanding of the action mode and binding specificity between pheromones and PBPs.

In recent years, the interactions between ligands and insect PBPs have been proposed based on the diversity of key residues. Many amino acids have been identified as the critical residues for ligands binding^19,25,32^. In moth species, the structure of BmorPBP/bombykol complex revealed that Ser56 forms a specific hydrogen bond between bombykol and BmorPBP^19^, and in *A. polyphemus*, Asn53 had been confirmed to be the key site in specific recognition of acetate^25^. Besides, the structure of LUSH/cVA complex in *Drosophila melanogaster* showed that cVA forms two polar interactions with Ser52 and Thr57 in the binding pocket^32^.

The cotton bollworm*, Helicoverpa armigera*, is one of the most serious agriculture pests worldwide and cause great damage to cotton and other crops^33^. This insect utilize *Z*11-16:Ald and *Z*9-16:Ald as the primary components of the pheromone blend^3^. Previously, three PBP genes, *HarmPBP1-3* have been identified and the results of fluorescence-binding assay revealed that HarmPBP1 equally bind the two principal pheromone components with strong affinities^34,35^. HarmPBP1 may play key roles in the pheromone perception of *H*. *armigera*. In the present study, we built a 3D model of the HarmPBP1 structure to predict the potential binding sites by homology modeling and molecular docking. The binding roles of these predicted residues were further investigated by site-directed mutagenesis and fluorescence binding assays. This work will help to deeply understand the interaction between HarmPBP1 and sex pheromone components in *H.armigera*.

## Results

### Expression of recombinant HarmPBP1

**Figure 1:**
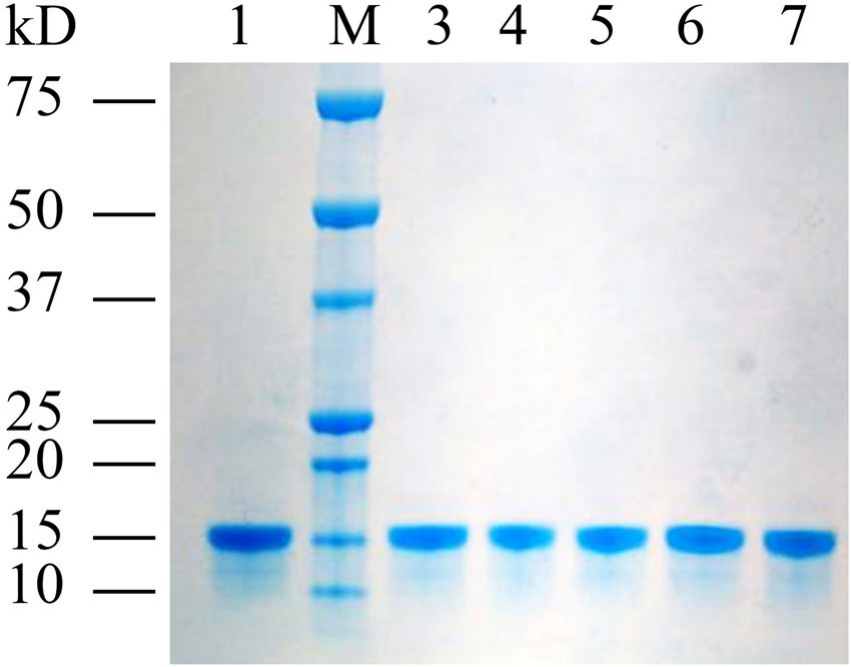
Sodium dodecyl sulphate polyacrylamide gel electrophoresis analysis of recombinant protein HarmPBP1 and mutant proteins. 1: wild-type HarmPBP1; 2: protein molecular weight marker; 3: mutant F12A; 4: mutant F36A; 5: mutant W37A; 6: mutant F119A; 7: mutant Q64A.

The coding region of HarmPBP1 was sub-cloned into an *E. coli* expression vector pET-32a/TEV and confirmed by PCR and sequencing. The protein expression was induced for 12 h by adding IPTG (1.0 mM) into the cell culture. The induced and non-induced cells were solicited and the crude inclusion body and supernatant were analyzed by SDS-PAGE. It was found that the recombinant HarmPBP1 was expressed in both supernatant and inclusion body. Then, the supernatant was collected and purified by His-Trap affinity columns (GE Healthcare, USA) followed by removal of the his-tag with TEV Protease. SDS-PAGE analysis indicated that the molecular weight of the final purified HarmPBP1 was about 15kD (Fig. 1), which is consistent with the theoretical molecular weight calculated by a computer pI/Mw online program (http://web.expasy.org/compute_pi/).

### 3-D structure modeling and molecular docking

**Figure 2:**
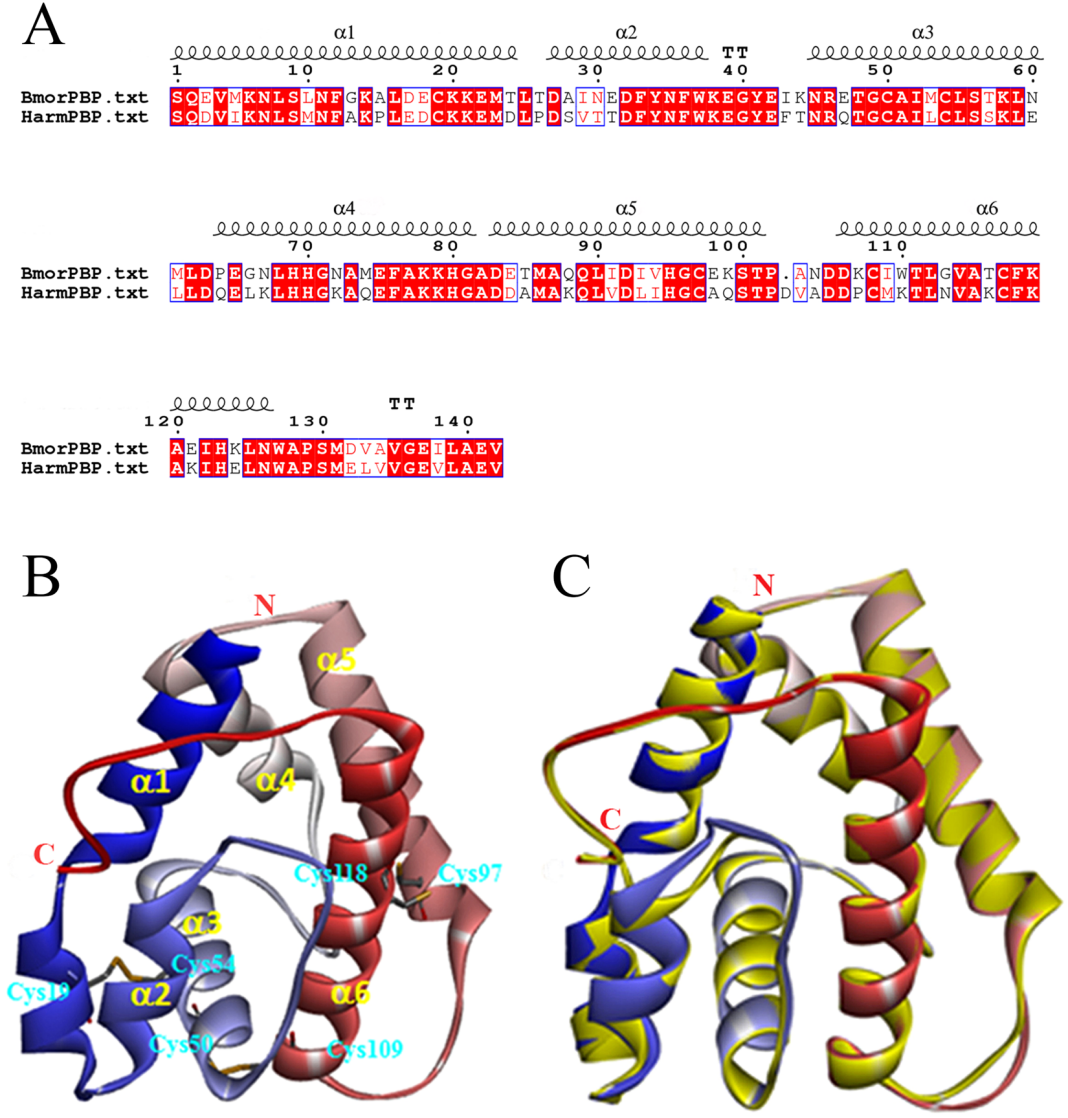
3-D structure model of HarmPBP1. (**A**) Sequence alignment of HarmPBP1 and BmorPBP. (**B**) Predicated 3-D model of HarmPBP1. Helices, N-terminal (N) and C-terminal (**C**) are labelled. Disulphide bridges are coloured yellow. (**C**) The alignment plot of the target protein HarmPBP1 and the template protein BmorPBP (yellow).

From a BLAST search in the Protein Data Bank (PDB), four structurally determined OBPs, *Bombyx mori* PBP (BmorPBP), *Amyelois transitella* PBP (AtraPBP1), *Antheraea polyphemus* PBP (ApolPBP) and *Bombyx mori* OBP (BmorGOBP2) were selected to share sequence similarities with HarmPBP1. The total sequence identity between the target protein (HarmPBP1) and the template protein (BmorPBP) is 67% (Fig. 2A). Thus, to guarantee the quality of the homology model, BmorPBP with the high level of sequence identity was used as a template to construct the 3D structure of HarmPBP1.

The overlap between 3D model of HarmPBP1 and template showed a high similarity of 0.828, which revealed that the overall conformation of target protein is very similar to the template (Fig. 2B,C). The predicted 3D structure demonstrated that HarmPBP1 is a “classical PBP”. Six α-helices were located between residues Ser1-Asp24 (α1), Asp27-Trp37 (α2), Asn45-Glu60 (α3), Gln64-Gly81 (α4), Asp83-Thr101 (α5), and Asp107-Asn127 (α6). Four antiparallel helices (α1, α4, α5 and α6) converge to form the hydrophobic binding pocket. The converging ends of the helices formed the narrow end of the pocket, and the opposite end of the pocket is capped by α3 (Fig. 2B). Disulphide bonds and helix–helix packing enforce the organization of the helices. Three pairs of disulfide bridges are observed between Cys19-Cys54, Cys50-Cys109, and Cys97-Cys118 (Fig. 2B). In this model, most of the amino acid residues that formed the pocket were hydrophobic, such as phenylalanine, tryptophan, alanine, valine, leucine, and isoleucine.

To further investigate the potential key residues in HarmPBP1, *Z*11-16:Ald and *Z*9-16:Ald were selected to dock with the 3D model. The docking results showed that both the two ligands are consistent in orientation, and highly overlapped in the same tunnel of binding pocket. Moreover, the oxygen of *Z*11-16:Ald and *Z*9-16:Ald are located in the similar position of binding cavity (Figure S1).

**Figure 3:**
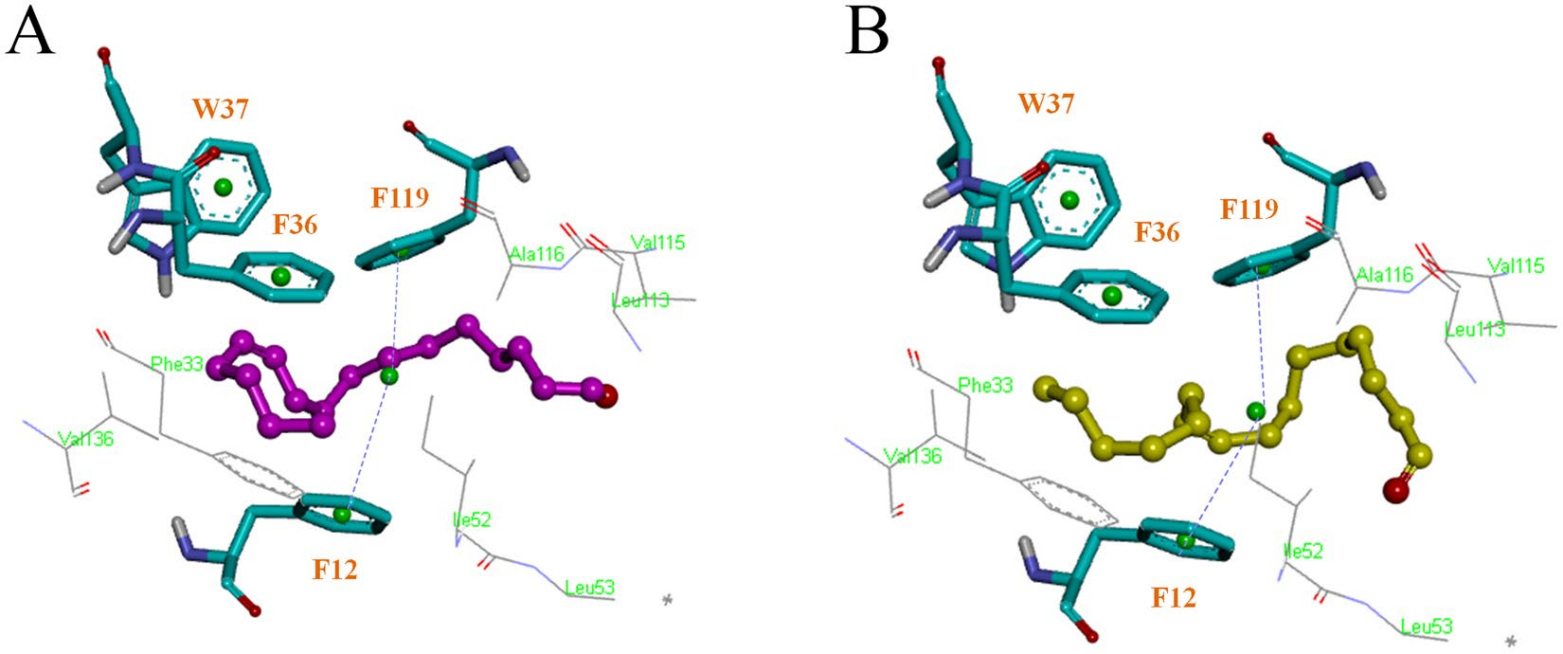
Docking results of *Z*11-16:Ald with the model (**A**), and *Z*9-16:Ald with the model (**B**).

Widely hydrophobic interactions have been observed as the main linkage between HarmPBP1 and the two aldehydes. All the hydrophobic residues included phenylalanine12 (Phe12), phenylalanine33 (Phe33), phenylalanine36 (Phe36), tryptophan37 (Trp37), Isoleucine52 (Ile52), Leucine53 (Leu53), Leucine113 (Leu113), Valine115 (Val115), Alanine116 (Ala116), phenylalanine119 (Phe119), and Valine136 (Val136) with less than 7.0 Å distances to *Z*11-16:Ald and *Z*9-16:Ald (Fig. 3). Amongst of these residues, Phe12 and Phe119 showed a sandwich-like pose to locate the binding conformation of ligands. Such hydrophobic contacts were much favorable to the binding between protein and ligands due to the nonpolar aromatic ring of Phe residue. Trp37 and Phe36 also provided certain nonpolar binding effects on the ligands with different sidechain.

### Site-directed mutagenesis and binding characterization of mutants

Based on the 3-D structure modeling and molecular docking described above, combined with an X-ray structure of the HarmPBP1/*Z*9-16:Ald complex (unpublished data), we predicted that four residues (Phe12, Phe36, Trp37, and Phe119) may play important roles in ligand binding. To verify the importance of such residues, the alanine scanning mutagenesis modeling have been performed, and the binding free energy for *Z*11-16:Ald and the wild-type (WT) or four mutants of HarmPBP1 were calculated (Table S1). Mutants F12A and F119A showed significant differences on binding to *Z*11-16:Ald from the WT. Meanwhile, W37A also showed a certain effect on the binding of *Z*11-16:Ald. However, the binding free energy of *Z*11-16:Ald and F36A changed only slightly compare to that of *Z*11-16:Ald and WT.

All the four residues were mutated to alanine, respectively, by using a site-directed mutagenesis kit. In addition, Gln64, a randomly selected residue on the loop between helices α3 and α4, was mutated to alanine as a control. The recombinant mutants F12A, F36A, W37A, F119A, and Q64A were expressed and purified as described above. The purified proteins were also checked by SDS-PAGE (Fig. 1). It was showed that the expression levels of mutants were apparently the same as that of wild-type HarmPBP1.

Fluorescence binding assays were performed in a reaction system at pH7.4. Probed by 1-NPN, the maximum emission wavelengths of F12A, F36A, W37A, F119A and Q64A were in the range of 390–410 nm, which are similar to that of HarmPBP1 (400 nm). The dissociation constant (Kd) of F12A/1-NPN, F36A/1-NPN, W37A/1-NPN, F119A/1-NPN, Q64A/1-NPN, and HarmPBP1/1-NPN complexes were 2.1 ± 0.17, 4.05 ± 0.69, 1.87 ± 0.18, 1.96 ± 0.16, 2.39 ± 0.19 and 1.79 ± 0.14 μM, respectively (Figure S2). These results revealed that the Kd values of all mutants were closed to that of wild type HarmPBP1.

**Figure 4:**
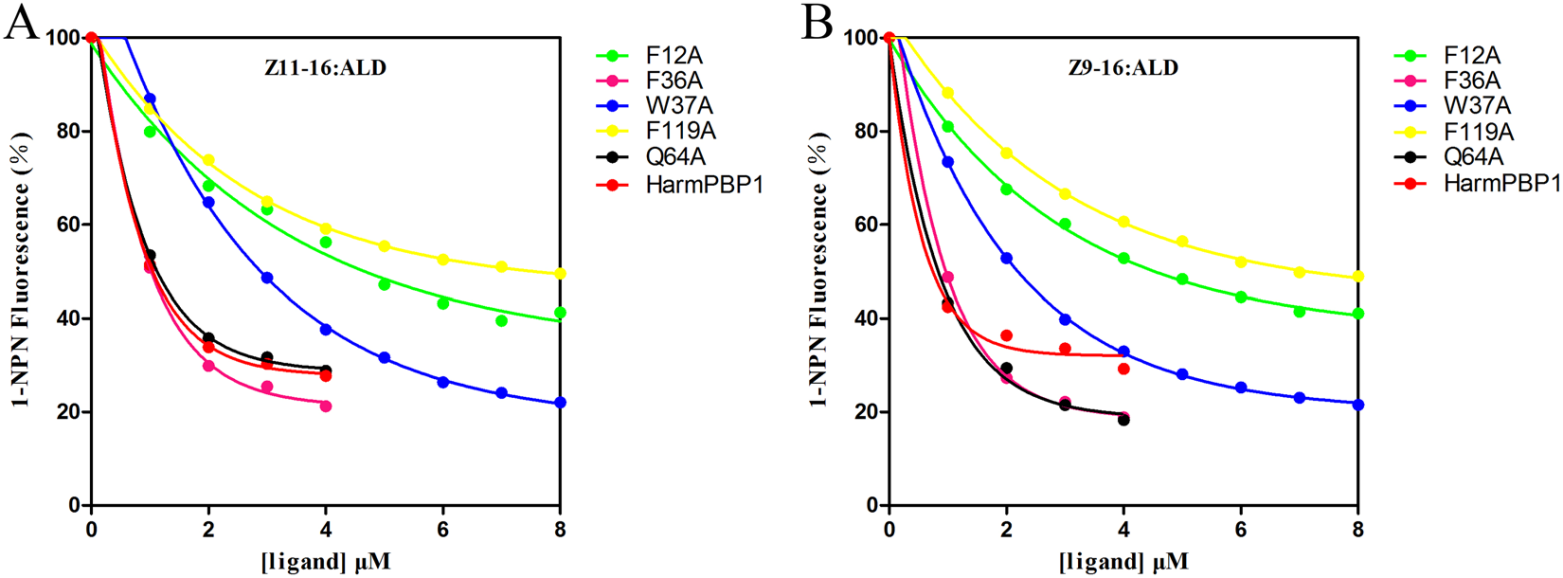
Competitive binding curves of *Z*11-16:Ald and *Z*9-16:Ald to the wild-type and mutants of HarmPBP1. (**A**) Binding curves of *Z*11-16:Ald to wild-type HarmPBP1 and all mutants. (**B**) Binding curves of *Z*9-16:Ald to wild-type HarmPBP1 and all mutants.

The affinities of all mutants with *Z*11-16:Ald and *Z*9-16:Ald were also investigated by fluorescence binding assays (Fig. 4). The results showed that compared to the wild-type HarmPBP1, each of the four mutants, F12A, F36A, W37A and F119A showed a different degree of decline in their binding capacities to the sex pheromone compounds, whereas, there was almost no change in the binding ability of Q64A with the two ligands. Three mutants, F12A, W37A and F119A had lower affinities to both *Z*11-16:Ald and *Z*9-16:Ald than that of F36A (Table 2). Compared to the wild-type HarmPBP1 (0.67 ± 0.05 μM to *Z*11-16:Ald and 0.56 ± 0.05 μM to *Z*9-16:Ald, separately), F119A showed the most dramatic decrease in binding capacity, with the dissociation constant (Ki) of 5.11 ± 0.47 μM to *Z*11-16:Ald and 4.61 ± 0.33 μM to *Z*9-16:Ald, respectively. F12A had a four to five fold decline in its affinity, with the Ki of 3.10 ± 0.13 μM to *Z*11-16:Ald and 3.06 ± 0.22 μM to *Z*9-16:Ald. W37A also showed decrease in binding ability to *Z*11-16:Ald and *Z*9-16:Ald, with Ki of 1.95 ± 0.08 μM and 1.49 ± 0.13 μM, respectively. However, compared to the wild-type HarmPBP1, F36A demonstrated only a slight decline in binding to *Z*9-16:Ald. Thus, three amino acids, Phe12, Trp37, and Phe119 in the binding pocket of HarmPBP1, should be the key residues which involved in the binding of *Z*11-16:Ald and *Z*9-16:Ald.

**Table 2:** Binding abilities of HarmPBP1 and mutants to *Z*11-16:Ald and *Z*9-16:Ald.

Proteins	*Z*11-16:Ald		*Z*9-16:Ald	
	IC_50_ (μM)	Ki (μM)	IC_50_ (μM)	Ki (μM)
HarmPBP1	1.03 ± 0.08	0.67 ± 0.05a	0.87 ± 0.08	0.56 ± 0.05a
F12A	4.69 ± 0.33	3.10 ± 0.13c	4.63 ± 0.27	3.06 ± 0.22c
F36A	1.04 ± 0.06	0.76 ± 0.08a	0.98 ± 0.15	0.72 ± 0.11a
W37A	2.91 ± 0.12	1.95 ± 0.08b	2.22 ± 0.19	1.49 ± 0.13b
F119A	7.66 ± 0.56	5.11 ± 0.47d	6.91 ± 0.49	4.61 ± 0.33d
Q64A	1.12 ± 0.21	0.77 ± 0.13a	0.84 ± 0.06	0.58 ± 0.03a

Data represent the mean values ± S.E.M of three independent replicates. Different letters within the same column mean that the values were significantly different (*P* < 0.05). IC_50_, ligand concentration displacing 50% of the fluorescence intensity of the protein/N-phenyl-1-naphthylamine complex; Ki, dissociation constant.

## Discussion

PBPs are known to bind and transport hydrophobic pheromone molecules across the sensillum lymph to PRs, and enhance the sensitivity of PRs to sex pheromones^13,14,16,36–39^. It was also reported that PBPs could specifically bind distinct pheromone components^11,15,40^, and such binding specificity was attributed to the spatial structure of proteins and ligands, especially their specific interactions^41^. As a result, clarifying the structure of insect PBPs should be helpful in better understanding of their binding mechanisms and biological roles in pheromone perception. In previous study, some crystal structures of lepidopteran PBPs have been solved by NMR or X-ray diffraction^19,21,22^. However, the structures of *H. armigera* OBP/PBPs are still lack.

Three PBPs of *H. armigera* have been reported in our previous study^35^. The results of fluorescence binding assay showed that HarmPBPs could specifically bind to different pheromone components of *H. armigera* ^34,35,42^. The main composition of *H. armigera* pheromone blend contain two hexadecane, *Z*11-16:Ald and *Z*9-16:Ald^43^. Both *Z*11-16:Ald and *Z*9-16:Ald own similar size of the carbon chain, and HarmPBP1 showed stronger affinities to these two aldehydes than to other minor components^34,35^. Therefore, we decided to predict the structure of HarmPBP1 by using 3D homology modeling, and *Z*11-16:Ald and *Z*9-16:Ald were selected as suitable ligands to dock with this structure.

From a BLAST research in the PDB, BmorPBP1 (1DQE) with most sequence similarity (67% identify) to HarmPBP1 was selected as the template to build a 3D homology structure of HarmPBP1. Subsequent docking results revealed that the binding cavity of HarmPBP1 is mainly formed by hydrophobic residues, and *Z*11-16:Ald and *Z*9-16:Ald are well overlapped in the binding packet (Figure S1). Widely hydrophobic interaction was observed to contribute the binding between protein and ligands, but no hydrogen action was found in this structure. Actually, although hydrogen bonds have been confirmed to be the primary link between proteins and ligands in several insect OBPs^44–47^, there are still some OBPs that only form hydrophobic interactions or van der Waals interactions^48,49^. In the docking structure of HarmPBP1, Phe12 and Phe119 are located on the two sides of the ligands, respectively, and the molecular plane of ligands is sandwiched by these two residues with their aromatic rings parallel (Fig. 3). Such sandwich-like pose contributes to solidify the binding conformation of ligands, so we suspected that Phe12 and Phe119 should be the important binding sites. Phe36 and Trp37 are close to the ligands, which may also play roles in the formation of hydrophobic interactions. Hence, we predicted that four active sites, Phe12, Phe119, Phe36 and Trp37, were possibly responsible for the ligand binding of HarmPBP1. The alanine scanning mutagenesis modeling was later performed to verify such prediction. The results showed that mutants F12A and F119A were of remarkable difference in binding to *Z*11-16:Ald from the wild-type of HarmPBP1, suggesting that these two residues of HarmPBP1 should be important on the ligand binding. W37A also showed a certain effect on the binding with *Z*11-16:Ald, indicating its potential contribution to the ligand binding. F36A demonstrated a slight change on the binding free energy of *Z*11-16:Ald, which suggested that this residue might not vital to the ligand binding.

Further site-directed mutagenesis and fluorescence binding assays were performed to characterize the binding abilities of the four mutants of HarmPBP1. A random mutation, Q64A was set as one of the control. The results of binding tests revealed that Q64A had no difference in affinity to *Z*11-16:Ald and *Z*9-16:Ald compared with the wild-type protein, which suggested that non-specific mutation could not affect the interactions between proteins and ligands. Both the single amino acid mutants, F12A and F119A could not efficiently bind to *Z*11-16:Ald and *Z*9-16:Ald. A possible explanation is that ligands cannot remain in the binding cavity due to the loss of the hydrophobic interactions between ligands and residues. Ligands are sandwiched by Phe12 and Phe119 with their aromatic rings, and such stable binding conformation was broken when any of these two residues was mutated to alanine. As a result, we suggested that Phe12 and Phe119 play the key roles in the ligand-binding of HarmPBP1. Mutant W37A showed a certain decrease in affinity to *Z*11-16:Ald and *Z*9-16:Ald due to the changes of hydrophobic interaction between the mutant and ligands. Thus, W37 is also an important binding site of HarmPBP1. Another mutant F36A, however, showed nearly no change in its binding ability to *Z*11-16:Ald and *Z*9-16:Ald. Therefore, we suspected that Phe36 may not be involved in the binding with *Z*11-16:Ald and *Z*9-16:Ald, or may participate in the binding with other ligands. All the four residues are highly conserved in lepidopteran PBPs and most GOBPs^19,25,35^, but only Phe12 and Phe119 contribute significantly to bind with the *Z*11-16:Ald and *Z*9-16:Ald. Interestingly, these two residues also play important roles in the binding process between BmorPBP1 and Bombykol^19^. Moreover, in SlitOBP1, the mutants of Phe12 and Phe118 result in lower docking scores to all tested chemicals in the simulation of site-direct mutagenesis, and the recombinant mutant Phe12 could not bind to all the ligands which exhibit good affinities to the wild-type protein^50^. Such results suggest that some conserved hydrophobic residues, such as Phe12 and Phe119, may be responsible for non-specific binding among different lepidopteran OBPs. On the other hand, strictly conserved Phe36 had been confirmed to be the key residue of LdisPBP1 in binding with its pheromone and analogues^51^. However in the current study, the affinity of mutant F36A to *Z*11-16:Ald and *Z*9-16:Ald showed nearly no change compared with the wild-type protein. In view of such difference, we speculated that beside the amino acids which contribute to non-specific binding, some other residues should be the key sites in binding with specific components in lepidopteran OBPs. And it is important and interesting to further clarify such functional difference between the conserved residues in the binding pocket.

Our data indicated that multiple hydrophobic interactions play the key roles in the ligand binding of HarmPBP1. It was also revealed that besides the NMR or X-ray diffraction of protein–ligand complexes, molecular docking and the mutant binding assay could be a potential and effective tool to further analyze the molecular mechanisms of ligand-protein interactions. Moreover, the results of this study may serve as a foundation for future studies on integrated pest management through manipulating the pheromone detection of target insects.

## Methods

### Insects

A colony of *H. armigera* was maintained in the laboratory of the Institute of Plant Protection, Chinese Academy of Agricultural Sciences. Larvae were reared on an artificial diet, and the conditions were maintained at 26 ± 1 °C, 60% ± 5% RH, and L 14 h: D 10 h. After emergence, adult moths were fed with 10% honey solution. Antennae were removed from three days old male moths and were immediately stored in liquid nitrogen till to use.

### RNA extraction and cDNA synthesis

Total RNA was isolated from antennae samples by SV Total RNA Isolation System (Promega, Madison, USA) following the manufacturer’s protocol. The integrity of the RNA was checked by using 1.2% agarose gel electrophoresis and quantified using a ND-1000 spectrophotometer (NanoDrop, Wilmington, DE, USA) at OD260 nm. The high concentration (>800 ng/μL) of the total RNA showed that the high quality of the RNA sample meet the standard of reverse transcriptase reaction. The first strand cDNA was synthesized using the SuperScript^TM^ III Reverse Transcriptase System (Invitrogen, Carlsbad, CA, USA).

### Expression and purification of recombinant HarmPBP1

The full sequence of *HarmPBP1* was identified from *H. armigera* antennal cDNA library in our previously work^42^. The sequence encoding mature *HarmPBP1* was amplified by PCR with gene-specific primers (Table 1). The PCR product was purified and sub-cloned into pGEM-T vector (Promega, Madison, USA). Target sequence was excised with *Nco* I and *Hind* III and then cloned into pET-32a/TEV vector (Novagen, Germany) with T4 DNA ligase. The correct recombinant plasmid pET/HarmPBP1 was transformed to BL21 (DE3) competent cells. Cells were incubated at 37 °C until OD_600_ reached 0.6–0.8, and the proteins were expressed after induction with 0.2 mM IPTG for 12 h. Cells were harvested by centrifugation at 7000 rpm for 20 min, and precipitate was re-suspended with 1 × phosphate-buffered saline (PBS). After ultrasonic, cells were centrifugalized at 16000 rpm for 20 min, then inclusion bodies and supernatant was collected and checked by 15% SDS-polyacrylamide gel electrophoresis (SDS-PAGE) analysis. The supernatant was filtered with a 0.22 μm ultrafiltration and purified by two rounds of Ni ion affinity chromatography (GE Healthcare,USA), and the His-tag was removed with Tobacco Etch Virus (TEV) protease (GenScript, Nanjing, China). The highly purified proteins were desalted through extensive dialysis. The size and purity of recombinant HarmPBP1 were confirmed by 15% SDS-PAGE analysis.

**Table 1:** Primers used in this study.

Primer name	Sequence (5′-3′)
***For recombinant proteins expression***	
HarmPBP1–forward	GGCCATGGCGTCGCAAGATGTTATTA^a^
HarmPBP1- reverse	GGAAGCTTTTAGACTTCGGCCAAG^a^
***For site-directed mutagenesis***	
F12A-forward	CCTCTCTATGAATGCCGCTAAGCCCTTAG^b^
F12A-reverse	CTAAGGGCTTAGCGGCATTCATAGAGAGG^b^
F36A-forward	CTTCTACAACGCCTGGAAGGAAGGC^b^
F36A-reverse	GCCTTCCTTCCAGGCGTTGTAGAAG^b^
W37A-forward	CTACAACTTCGCGAAGGAAGGCTAC^b^
W37A-reverse	GTAGCCTTCCTTCGCGAAGTTGTAG^b^
F119A-forward	GGCCAAGTGCGCCAAGGCCAAGATA^b^
F119A-reverse	TATCTTGGCCTTGGCGCACTTGGCC^b^
Q64A-forward	GCTACTGGACCAGGAGCTCAAGC^b^
Q64A-reverse	GCTTGAGCTCCTGGTCCAGTAGC^b^

^*a*^“__”represent the restriction sites, ^*b*^“__” represent the mutation sites.

### 3D structure modeling and molecular docking

The 3D model of HarmPBP1 were built with a template of BmorPBP1 (1DQE) by using On-line Swiss-model software (https://www.swissmodel.expasy.org/). The binding cavity was predicted with an automobile mode by SYBYL 7.3 software. The molecular conformations of *Z*11-16:Ald and *Z*9-16:Ald were constructed by Sketch mode and optimized using the Tripos force field and Gasteiger-Hückel charge. The Surflex-Dock module of SYBYL 7.3 was employed to perform the molecular docking modeling^52^. The binding cavity was set as “Automatic” and the Total Score was used to evaluate the binding affinity between ligands and protein^53^. All molecular modeling between putative HarmPBP1 protein and ligands were conducted on the Silicon Graphics® (SGI) Fuel Workstation (Silicon Graphics International Corp., CA, USA).

### Simulation of Site-directed mutagenesis and the expression of mutants

The alanine scanning mutagenesis modeling were performed by the AMBER 14 package^54^ to verify the predicted key binding sites, and the binding free energy between the active site and *Z*11-16:Ald was calculated by the MM-GBSA method^55^.

Four mutations of HarmPBP1, F12A (mutating phenylalanine to alanine at position 12), F36A (mutating phenylalanine to alanine at position 36), W37A (mutating tryptophan to alanine at position 37) and F119A (mutating phenylalanine to alanine at position 119) were generated by using the Quick-change lightning site-directed mutagenesis kit (Stratagene, USA), and a random mutation, Q64A (mutating glutanine to alanine at position 64) was set as control. The pGEM-T Easy/HarmPBP1 construct was used as template, and the specific primers designed for mutations were also listed in Table 1. The PCR conditions were 95 °C for 30 s, followed by 18 cycles of 95 °C for 30 s, 60 °C for 1 min, 68 °C for 4 min. Valid mutants were sub-cloned into pGEM-T easy vector (Promega, USA). Same expression vector and competent cells were used as the HarmPBP1. The recombinant mutant protein prokaryotic expression and purification were conducted as mentioned above.

### Fluorescence binding assays

Fluorescence binding assays were conducted on the F-380 fluorescence spectrophotometer (Gangdong Sci. & Tech, Tianjin, China) in a 1-cm light path quartz cuvette to further investigate the binding abilities of the principal pheromone components of *H. armigera*, *Z*11-16:Ald and *Z*9-16:Ald, to mutants. The fluorescent probe N-phenyl- 1-naphthylamine (1-NPN) was dissolved in methanol to yield a 1 mM stock solution. Both of the excitation and emission slit widths were 10 nm. Fluorescence of 1-NPN was excited at 337 nm and the emission spectra were recorded between 390 and 490 nm. *Z*11-16:Ald and *Z*9-16:Ald were purchased from Sigma-Aldrich (purity >98%). All chemicals used in this study were dissolved in HPLC purity grade methanol. Fluorescence measurements were performed according to Gu *et al*.^11^. Dissociation constants of the competitors were calculated from the corresponding IC_50_ (the ligand concentration displacing 50% of the NPN fluorescence intensity of the HarmPBP1/1-NPN complex) values, using the equation: Ki = [IC_50_]/(1 + [1-NPN]/K_1-NPN_), where [1-NPN] is the free concentration of 1-NPN and K_1-NPN_ is the dissociation constant of the HarmPBP1/1-NPN complex.

## Electronic supplementary material

Supplementary information
