# Supplementary material for: Key site residues of pheromone-binding protein 1 involved in interacting with sex pheromone components of Helicoverpa armigera
Source: Sci Rep. 2017 Dec 4;7:16859. doi: 10.1038/s41598-017-17050-5 (PMC5715060; doi:10.1038/s41598-017-17050-5)
Supplement: Supplementary file 1 — Supplementary information [file 41598_2017_17050_MOESM1_ESM.pdf]

Key site residues of pheromone-binding protein 1 involved in interacting with sex pheromone components of *Helicoverpa armigera*

Kun Dong[1, 2, †], Hong-Xia Duan[3, †], Jing-Tao Liu[1], Liang Sun[1,4], Shao-Hua Gu[1], Ruo-Nan Yang[1], Khalid Hussain Dhiloo[1,5], Xi-Wu Gao[2], Yong-Jun Zhang[1, *], Yu-Yuan Guo[1]

[1] State Key Laboratory for Biology of Plant Diseases and Insect Pests, Institute of Plant Protection, Chinese Academy of Agricultural Sciences, Beijing, 100193, China

[2] Department of Entomology, China Agricultural University, Beijing 100193, China

[3] College of Science, China Agricultural University, Beijing 100193, China

[4] Key Laboratory of Tea Biology and Resources Utilization, Ministry of Agriculture, Tea Research Institute, Chinese Academy of Agricultural Sciences, Hangzhou, 310008, China

[5] Department of Entomology, Faculty of Crop Protection, Sindh Agriculture University Tandojam, Pakistan

[†] These authors contributed equally to this work.

[*] Corresponding author:

Yong-Jun Zhang PhD. State Key Laboratory for Biology of Plant Diseases and Insect Pests, Institute of Plant Protection, Chinese Academy of Agricultural Sciences, Beijing, 100193, China

E-mail: yjzhang@ippcaas.cn Tel.: +86 10 62815929; Fax: +86 10 62816631.

Table S1 The calculated binding free energy (ΔG) in vitro between *Z*11-16:Ald and the WT and different mutants of HarmPBP1.

Proteins	ΔG (kJ/mol)	ΔΔG
WT	-45.31	0
F12A	-39.28	6.03
F36A	-47.47	2.16
W37A	-41.06	4.25
F119A	-39.60	5.71

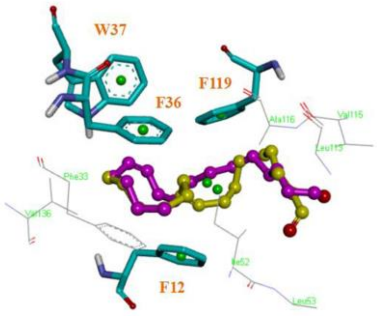

Figure S1 The overlap between *Z*11-16:Ald and *Z*9-16:Ald in the binding pocket of HarmPBP1. Purple, *Z*11-16:Ald; Yellow, *Z*9-16:Ald.

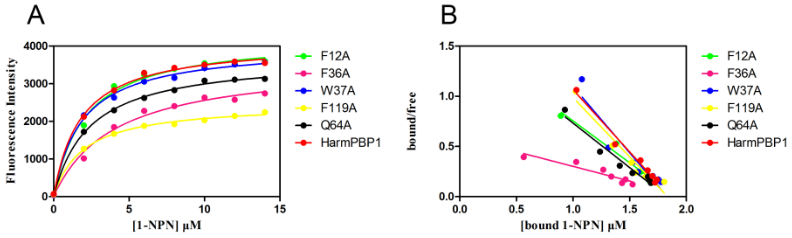

Figure S2 The binding curves and relative Scatchard plots of N-phenyl-1-naphthylamine (1-NPN) and HarmPBP1 as well as its mutants at pH 7.4.
